# Patterns of pediatric and adolescent female genital inflammation in China: an eight-year retrospective study of 49,175 patients in China

**DOI:** 10.3389/fpubh.2023.1073886

**Published:** 2023-09-04

**Authors:** Huihui Gao, Yuchen Zhang, Yanzheng Pan, Mengjia Zhao, Ye Qi, Mingming Zhou, Symphorosa S. C. Chan, Siyi Huang, Peige Song, Kun Tang, Liying Sun, Changzheng Yuan

**Affiliations:** ^1^Department of Pediatrics and Adolescent Gynecology, Children’s Hospital, Zhejiang University School of Medicine, National Clinical Research Center for Child Health, Hangzhou, China; ^2^School of Public Health, Zhejiang University School of Medicine, Hangzhou, China; ^3^Department of Clinical Laboratory, Children’s Hospital, Zhejiang University School of Medicine, National Clinical Research Center for Child Health, Hangzhou, China; ^4^Department of Obstetrics and Gynaecology, The Chinese University of Hong Kong, Shatin, China; ^5^Vanke School of Public Health, Tsinghua University, Beijing, China

**Keywords:** pediatric and adolescent gynecology, girls, genital inflammation, disease pattern, vulnerable population, pathogens

## Abstract

**Background:**

Genital inflammation is one of the most frequent clinical complaints among girls, which was easily overlooked by the general public. This study aimed to investigate the patterns and epidemiological characteristics of pediatric and adolescent female genital inflammation in China.

**Methods:**

A retrospective observational study (2011 to 2018) was conducted among all female patients under the age of 0–18 years at the Department of Pediatric and Adolescent Gynecology of The Children’s Hospital, Zhejiang University School of Medicine. Data were collected from the electronic medical records. The abnormal vaginal discharge of patient was collected for microbiological investigation by bacterial and fungal culture. Descriptive analysis was conducted to evaluate the genital inflammation pattern and epidemiological characteristics, including age, season, and type of infected pathogens.

**Results:**

A total of 49,175 patients met the eligibility criteria of genital inflammation and 16,320 patients later came to the hospital for follow-up over the study period. The number of first-visit increased gradually from 3,769 in 2011 to 10,155 in 2018. The peak age of the first visit was 0–6 years old. Non-specific vulvovaginitis, lichen sclerosis, and labial adhesion were the top three genital inflammation. Among the top five potential common pathogens of vaginal infection, the prevalence of *Haemophilus influenzae* cases was the highest (31.42%, 203/646), followed by *Streptococcus pyogenes* (27.74%, 176/646), *Candida albicans* (14.09%, 91/646), *Escherichia coli* (8.51%, 55/646), and *Staphylococcus aureus* (6.35%, 41/636). The specific disease categories and pathogens of genital inflammation vary by age groups and season.

**Conclusion:**

Our study summarizes the pattern of pediatric and adolescent female genital inflammation over an 8-year period in China, emphasizing the need for more public awareness, healthcare services and research in this field.

## Introduction

Female genital inflammation is manifested by abnormal vaginal discharge, odor, itching, or discomfort and affects up to 75% of girls and women during their lifetime (1). It has been reported that genital inflammation is the most common gynecological disorder among pediatric and adolescent females (2, 3). However, as a frequent complaint among girls, genital inflammation is easily overlooked by the general public. Previous evidence showed genital inflammation in adults may lead to adverse outcomes, such as endometriosis, reproductive disorders, and even gynecologic malignancies (4, 5), which has received attention in adult women. Moreover, untreated and recurrent problems contribute to a severe disease burden such as increasing the incidence of HIV and female infertility (6, 7). This problem among girls influences future reproductive health and frequently causes great anxiety in children and their parents. Especially, for girls aged 0–18 who were the “future mothers,” their reproductive and psychological health deserves the attention of public health and clinical staff.

The patterns and characteristics of genital inflammation among girls differ from adults, due to the significant differences in hormone levels, lifestyle habits, and sexual behavior. For example, sexually transmitted infections, which are more common in adult females, are rare in adolescent females and young females in China (8). These unique characteristics indicate that this population has been neglected and needs more research to reveal the patterns and epidemiological characteristics and understand how girls have been impacted by genital inflammation. The information can reduce misdiagnosis and inappropriate treatment. There is substantial room to improve sexual and reproductive health (SRH) services (9).

Recently, several studies have focused on genital inflammation while most of them were mainly conducted in western countries with small sample sizes (10, 11). Therefore, our study performed a retrospective analysis of data from pediatric gynecology outpatients from the Children’s Hospital of Zhejiang University School of Medicine for eight consecutive years to understand the characteristics and distribution of genital inflammation in Chinese girls. This study aims to increase the understanding of genital inflammation among girls in both local and global contexts as well as provide evidence for the subsequent development of SRH services for young girls.

## Methods

### Study design

We assessed the data of all outpatients aged 0–18 treated in the Pediatric and Adolescent Gynecology (PAG) Department in The Children’s Hospital, Zhejiang University School of Medicine, from January 2011 to December 2018. Based on the hospital outpatient service system, all electronic medical records (EMRs) of our outpatients with genital inflammation were extracted. EMRs of outpatients consisted of the basic demographic information of patients, the history of present illness, physical examination, and auxiliary examination, recorded the details of the clinician’s inquiry and observation of the patients’ statuses. The research roadmap was showed in Supplementary Figure 1. The study was approved by the Human Subjects Committees of the Children’s Hospital, Zhejiang University School of Medicine (approval number 2019-IBR-103).

### Study population

The inclusion criteria for the study were as follows: ① outpatients at the PAG clinic who were under the age of 18 between 2011 and 2018; ② Girls who were diagnosed as non-specific vulvovaginitis, bacterial vulvovaginitis, vulvovaginal candidiasis (VVC), sexually transmitted infections (STIs), labial adhesion, lichen sclerosis, vulvar abscess, and vaginal foreign body.

Exclusion criteria: ① Patients with congenital immune deficiency or diabetes; ② History of sexual life or sexual violence; ③ “Diagnosis time,” “first diagnosis” and other items missing If one of the above conditions is satisfied, the patient is excluded.

### Identification of microorganisms

The abnormal vaginal discharges of participants were collected according to our previous study (12). The patients were examined in lithotomy position. The labia were separated, and a swab was taken from the introitus or, if possible, the lower third of the vagina. All swabs were kept in sterile tubes before being inoculated on plates in 1 h. Culture plates included blood agar plate (Columbia base agar containing 5% defibrinated sheep blood, BioMerieux, France), Haemophilus selective medium plate (BioMerieux, France), and Neisseria gonorrhoea selective medium plate (BioMerieux, France), Sabouraud’s Medium with triphenyltetrazolium chloride (triphenyltetrazolium chloride, TTC; Bosai, China).

The blood agar plates and Haemophilus-selective medium plates were cultured in an incubator containing 5–8% carbon dioxide for 16–24 h. The N. gonorrhoea−selective plates were cultured in an air incubator for 48 h. Regarding fungal culture, samples were applied to Sabouraud’s Medium with TTC were cultured in an air incubator for 24–72 h at 37°C. Bacteria or fungi grown in the pure culture or as the dominant organism were classified as pathogens and were identified further. Organisms were identified with Gram positive identification card, Gram-negative identification card, or Neisseria−Haemophilus identification card in an automatic bacteria identification system (Vitek, BioMerieux, France) after Gram staining, catalase test, and oxidase test. *Hemolytic streptococci* were grouped by rapid latex test (BioMerieux, France), and *Streptococcus pneumoniae* isolates were also tested with optochin discs (Oxoid, England).

### Assessment of genital inflammation

In the study, genital inflammation in the PAG department was assessed based on the first diagnosis among patients at their first clinical visit. Referring to recent literature and textbook (8, 13–15), all newly diagnosed patients were categorized into eight major diagnoses: non-specific vulvovaginitis, bacterial vulvovaginitis, VVC, STIs, labial adhesion, lichen sclerosis, vulvar abscess, and vaginal foreign body. Patients with abnormal vaginal discharge were investigated pathogenic test by bacterial or fungal culture. The pathogenic results were also collected from patients with vaginal infections between 2015 and 2018 and divided into three categories: bacterial vulvovaginitis, VVC, and STIs. Aerobic vaginitis (AV), bacterial vaginosis (BV) belonged to bacterial vulvovaginitis.

### Covariates

Information on covariates, including age, date of diagnosis, date of birth, date of visit and residency, first diagnosis, visiting status (first visit or subsequent visit), and pathogen detection, were extracted from patients’ EMR. The demographic characteristics were also collected. Visit year, season, and month were derived from the date of diagnosis. Age was calculated based on the time interval between the date of birth and the date of diagnosis. Based on the WHO definition of children and adolescents, we divided girls aged 0–18 into three groups: children (pediatric,0–6 years old), pre-adolescence (prepuberty, 7–9 years old), and late adolescence (late puberty, 10–18 years old). In this study, genital inflammation in the PAG department was assessed based on the first diagnosis at their first clinical visit. When patients have more severe symptoms or recurrence symptoms, they visited the PAG department for follow-up consulting. The interval time for multi-visit patients was the number of days between the date of the first visit and the last visit; the average revisits time interval for repeat patients is the total interval divided by the number of follow-up visits. One individual extracted all EMR information. The data was then independently reviewed by a second and third individual.

### Statistical analysis

Descriptive analysis was conducted to evaluate the pattern of genital inflammation in children and teenagers by age, year, and season. Continuous variables were expressed as mean and standard deviation (SD), median, and quartile (Q1, Q3) according to whether the data were normally distributed. Categorical variables were presented as the number of cases and the percentage. The χ^2^ test was used to detect whether there were significant statistical differences in the distribution of outpatients by three different age groups under some basic demographic characteristics. The Kruskal Wallis test was applied to data with skewed distributions across multiple age groups such as interval time. The comparison of differences in the patterns including genital inflammation issues and pathogens across age group, year, and season were evaluated using the R × C χ^2^ test of the composition ratio of multiple samples and Fisher’s exact probability method when the R × C χ^2^ test was not the most effective analysis. *p* value < 0.05 was considered statistically significant. All analyses were conducted using SAS software, version 9.4 (SAS Institute Inc., Cary, NC) and R (version 4.2.3). All graphs were drawn using GraphPad Prism 8.4.3.

## Results

### Basic characteristics of the study population

A total of 49,175 patients with genital inflammation visited the PAG department over 8 years, and the total number of visits was 76,807. During the study period, the number of PAG patients that attended for the first visit increased gradually from 3,769 in 2011 to 10,155 in 2018. Table 1 shows the basic characteristics of patients at the first clinical visit during the study period. Patients’ ages ranged from 0 to 18 with a mean ± SD of 5.03 ± 3.16 years old; 68.74% of the participants were aged 0–6 years, 22.23% were aged 7–9 years, and 9.03% were aged 10–18 years. Patients came from Zhejiang province in 94.21% of cases, with 62.05% hailing from Hangzhou, where the hospital was situated. 16,087 patients had two or more visits within 8 years. The average number of follow-up visits for them was 1.72, and the median interval between each follow-up visit was 24 days.

**Table 1:** Basic characteristics of pediatric and adolescent gynecology outpatients with genital inflammation at first visits, overall and by age group.

Characteristics	Overall (*N* = 49,175)	Age 0–6 (*N* = 33,804)	Age 7–9 (*N* = 10,931)	Age 10–18 (*N* = 4,440)	p*
Age in years, mean (SD)	5.03 (3.16)	3.32 (1.90)	7.82 (0.79)	11.20 (1.45)	
Follow-up times, *N* (%)^#^					<0.0001
1	10,144 (63.06)	6,721 (66.92)	2,489 (58.40)	934 (52.41)	
2	3,370 (20.95)	2,050 (20.41)	917 (21.52)	403 (22.62)	
≥3	2,573 (15.99)	1,272 (12.67)	856 (20.08)	445 (24.97)	
Interval time, days, median (Q_1_, Q_3_)^#^					
First and last visit	36 (7,295)	29 (7,223)	56 (7,419)	60 (11,479)	<0.0001
Visit-to-visit	24 (7,156)	21 (7,130)	32 (7,208)	31 (8,197)	<0.0001
Year					<0.0001
2011–2014	18,217 (37.05)	12,149 (35.94)	4,236 (38.75)	1,832 (41.26)	
2015–2018	30,958 (62.95)	21,655 (64.06)	6,695 (61.25)	2,608 (58.74)	
Zhejiang province^##^	28,820 (94.21)	20,274 (94.80)	6,082 (92.63)	2,464 (93.37)	
Hangzhou city^###^	17,838 (62.08)	13,147 (64.99)	3,380 (55.74)	1,311 (53.38)	
Season					<0.0001
Spring	8,895 (18.09)	5,681 (16.81)	2,192 (20.05)	1,022 (23.02)	
Summer	12,611 (25.64)	8,961 (26.50)	2,648 (24.22)	1,002 (22.57)	
Fall	14,805 (30.11)	9,457 (27.98)	3,762 (34.42)	1,586 (35.72)	
Winter	12,864 (26.16)	9,705 (28.71)	2,329 (21.31)	830 (18.69)	

The number of outpatients with follow-up is 16,320. p* values are derived by χ^2^ test, except for interval time, where the Kruskal Wallis test was used.

# The number of outpatients with follow-up is 16,087.

## There is a missing value, and the missing value is 18,584.

### The missing value for patient analysis in Zhejiang cities is 70.

### Genital inflammation patterns overall and by age groups

The patterns of eight categories of genital inflammation by age group were shown in Table 2. There were significant differences in the age distribution of patients with the eight subgroups of genital inflammation patterns (*p* < 0.05). In general, non-specific vulvovaginitis accounted for 61.51% (30,247/49,175) of the first visit, followed by lichen sclerosis (9,721, 19.77%) and labial adhesion (4,260, 8.66%), bacterial vulvovaginitis (3,750, 7.63%), VVC (713, 1.45%), vaginal foreign body (331, 0.67%), vulvar abscess (102, 0.21%), STIs (51, 0.10%), respectively. The median age for various subtypes of patients with genital inflammation can be found in Figure 1A. The vaginal infections include bacterial vulvovaginitis, VVC and STIs according to the etiology of vaginal secretions, of which bacteria had the highest number of patients (*n* = 3,750) with a median onset age of 7(Q1 = 5, Q3 = 9). The next was VVC with a median age of 9(Q1 = 6, Q3 = 11), and the last was STIs with a median age of 5. Children 0–6 years old had a greater frequency of non-specific vulvovaginitis, with a median onset age of 5. With a median onset age of 1 and 4, lichen sclerosis and labial adhesion were most prevalent in children aged 0 to 6. Before age 9, the vaginal foreign body was often observed in girls. Children aged 0–6- and 10–18-years old adolescent females both had a reasonably high prevalence of vulvar abscess, with proportions of 49.02 and 30.39%, respectively.

**Table 2:** The proportion of specific genital inflammation issues in PAG department and by age group (*p* < 0.0001).

Subtypes, *N* (%)	Overall^*^	Age 0–6^#^	Age 7–9^#^	Age 10–18^#^	*p*(χ^2^)
Non-specific vulvovaginitis	30,247 (61.51)	19,810 (65.49)	7,543 (24.94)	2,894 (9.57)	<0.0001(5143.5084)
Lichen sclerosis	9,721 (19.77)	7,775 (79.98)	1,481 (15.24)	465 (4.78)	
Labial adhesion	4,260 (8.66)	4,207 (98.76)	37 (0.87)	16 (0.37)	
Bacterial vulvovaginitis	3,750 (7.63)	1,518 (40.48)	1,517 (40.45)	715 (19.07)	
Vulvovaginal candidiasis	713 (1.45)	220 (30.86)	194 (27.21)	299 (41.94)	
Vaginal foreign body	331 (0.67)	188 (56.80)	124 (37.46)	19 (5.74)	
Vulvar abscess	102 (0.21)	50 (49.02)	21 (20.59)	31 (30.39)	
STIs	51 (0.10)	36 (70.59)	14 (27.45)	1 (1.96)	

STIs: Sexually transmitted infections. *p <* 0.01 means there were significant differences in the distribution of outpatients under three different age groups and this difference was seen in at least two of the eight subtypes mentioned above. R × C χ^2^ test was used for comparison of the differences in age composition ratios of the eight subtypes.

* Percentage (%) is the proportion of each individual subtype in the genital inflammation pattern.

# Percentage (%) is the proportion of each individual subtype under different age groups.

**Figure 1:**
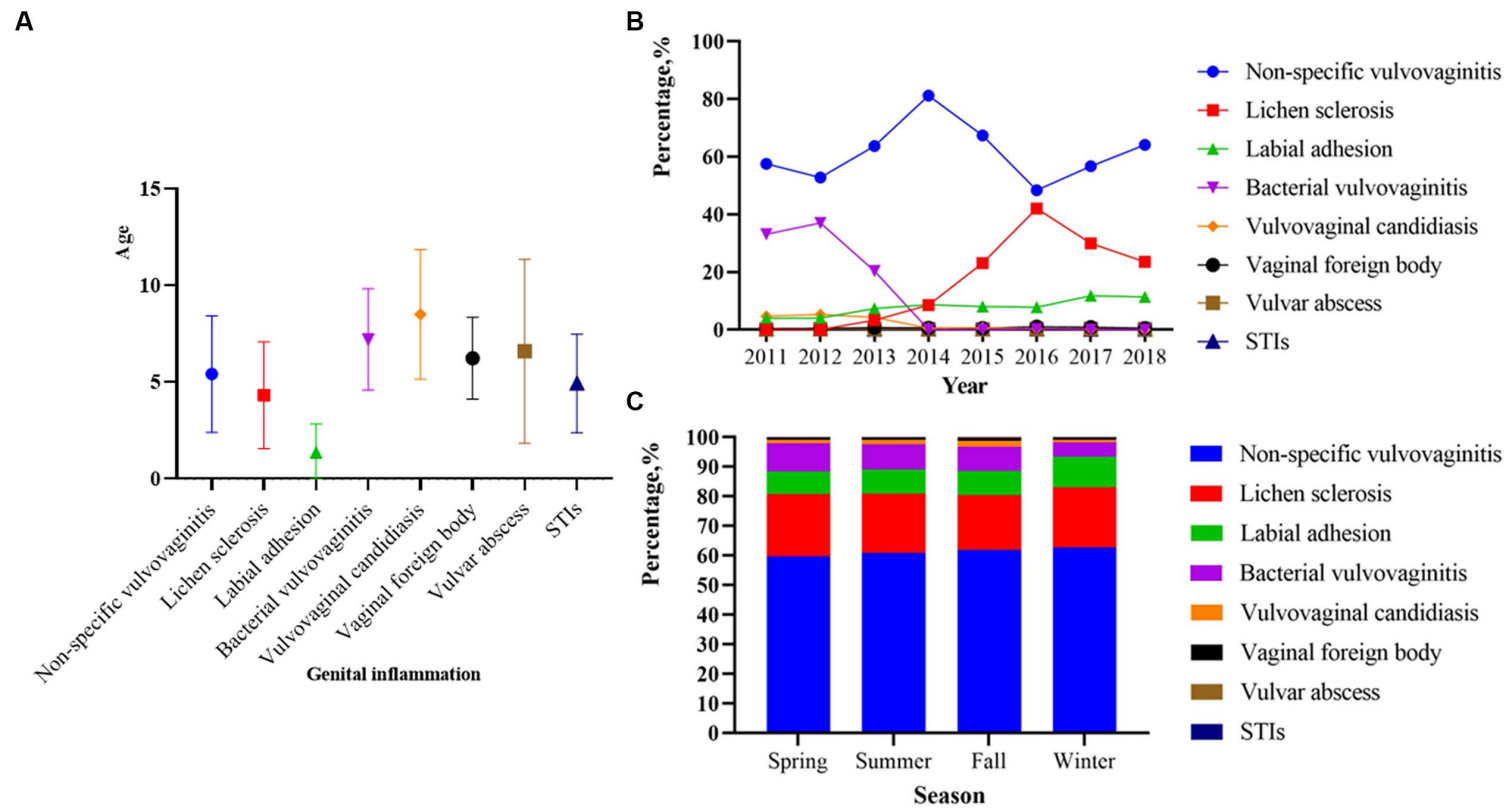
Age, year and season distribution of genital inflammation patterns among 49,175 outpatients from 2011 to 018 (*p* < 0.01). **(A)** Median age of onset corresponding to different genital inflammation patterns. **(B)** The percentage of genital inflammation patterns across different years (*p* < 0.01). **(C)** The percentage of genital inflammation patterns across different seasons (*p* < 0.01). *p* < 0.0001 in **(B)** represents a significant difference in the overall composition ratio of the above eight genital inflammation issues over the 8-year period. R × C χ^2^ test of composition ratios was used for comparing whether there was a difference in the composition ratio of eight issues in these years. *p* < 0.0001 in **(C)** represents a significant difference in the overall composition ratio of the above eight genital inflammation issues over four seasons. R × C χ^2^ test of composition ratios was used for comparing whether there was a difference in the composition ratio of eight issues in four seasons.

### Genital inflammation patterns by year and season

Between 2011 and 2018, the number of visits in the latter 4 years was nearly twice as high as in the first 4 years (Table 1). Of the 49,175 patients with genital inflammation, non-specific vulvovaginitis was the most common problem from 2011 to 2018 (Table 2), with the highest percentage of 81.17% in 2014 (Figure 1B). From 2011 to 2013, bacterial vulvovaginitis and VVC had high rates of vaginal infections (roughly 25 and 5%, respectively) but the proportion has declined since 2014. The number of STI cases was low, and the percentage fluctuated little from year to year, consistently not exceeding 1%. The prevalence of labial adhesion had largely been on an upward trend over the last 8 years, with the proportion of the genital inflammation pattern exceeding 10% after 2017. The percentage of lichen sclerosis was above 20% in 2015 and after and reached its highest percentage of 42.07% in 2016. The composition ratio of these eight disease subtypes varies across years in the overall (*p* < 0.05, Figure 1B).

The cumulative number of patients in the past 8 years in summer, fall, and winter was more than 10,000, and that in spring was less than 10,000 (Table 1). Figure 1C shows that the proportions of people with non-specific vulvovaginitis and lichen sclerosis were roughly evenly distributed across four seasons. Bacterial vulvovaginitis was more common in spring (839, 9.39%) and summer (1,086, 8.61%). VVC (305, 2.06%), STIs (20, 0.14%), vaginal foreign bodies (122, 0.82%), and vulvar abscesses (1,214, 8.20%) were more common in the fall. The labial adhesion increased in the winter (1,338, 10.40%) but bacterial vulvovaginitis significantly reduced in the winter (614, 4.77%). The composition ratios of these eight disease subtypes in seasons presented significant differences overall during the 8 years (*p* < 0.05, Figure 1C).

### The distribution of pathogens in patients with vaginal infections

Between 2015 and 2018, 666 cases completed pathogenic testing and detailed basic demographic information and 646 examinations were included in the statistical analysis. From the 4 years of data available on the top 10 potential common microbial pathogens, the prevalence of *Haemophilus influenzae* cases was the highest (203, 31.42%), followed by *Streptococcus pyogenes* (176, 27.74%), *Candida albicans* (91, 14.09%), *Escherichia coli* (*E. coli*; 55, 8.51%), *Staphylococcus aureus* (41, 6.35%), *Klebsiella pneumoniae* (12, 1.86%), *Streptococcus agalactiae* (8, 1.24%), *Streptococcus pneumoniae* (6, 0.93%), *Haemophilus parainfluenzae* (5, 0.77%), *Neisseria gonorrhoeae* (4, 0.62%). And the age distribution of patients within the 10 pathogen classifications was statistically different (*p* < 0.05, Table 3). *Haemophilus influenzae* represented the highest proportion of cases in the group aged 0–6 (152, 74.88%). In groups of 0–6 and 7–9, other pathogens with significant age peaks are *Streptococcus pyogenes* (90, 51.14% and 81, 46.02%), *Escherichia coli* (32, 58.18% and 21, 38.18%), *Staphylococcus aureus* (18, 43.90% and 21, 51.22%), and *Klebsiella pneumoniae* (4, 33.33% and 6, 50.00%). These pathogens above are uncommon in adolescents aged 10–18. However, *Candida albicans* are highly prevalent in girls aged 10–18 years (70, 76.92%), followed by girls aged 7–9 years (21, 23.08%).

**Table 3:** Age characteristic of patients with genital inflammation of top10 pathogens, overall and by age groups from 2015 to 2018 (*p* < 0.0001).

No	Pathogens, *N*(%)	Overall^*^	Age 0–6^#^	Age 7–9^#^	Age 10–18^#^	*p*
1	*Haemophilus influenzae*	203(31.42)	152(74.88)	49(24.14)	2(0.99)	0.0004998
2	*Streptococcus pyogenes*	176(27.74)	90(51.14)	81(46.02)	5(2.84)	
3	*Candida albicans*	91(14.09)	0	21(23.08)	70(76.92)	
4	*Escherichia coli*	55(8.51)	32(58.18)	21(38.18)	2(3.64)	
5	*Staphylococcus aureus*	41(6.35)	18(43.90)	21(51.22)	2(4.88)	
6	*Klebsiella pneumoniae*	12(1.86)	4(33.33)	6(50.00)	2(16.67)	
7	*Streptococcus agalactiae*	8(1.24)	2(25.00)	3(37.50)	3(37.50)	
8	*Streptococcus pneumoniae*	6(0.93)	5(83.33)	1(16.67)	0	
9	*Haemophilus parainfluenzae*	5(0.77)	3(60.00)	1(20.00)	1(20.00)	
10	Neisseria gonorrhoeae	4(0.62)	3(75.00)	1(25.00)	0	
	Others	45(6.97)	21(46.67)	16(35.56)	8(17.78)	

The number of cases of the top 10 pathogens mentioned above was 646, and the number of cases of other pathogens was 45, which was not reflected in the table. *p* < 0.01 means the age distribution of the above pathogens was statistically different in at least two groups of patients with different pathogens. R × C fisher’s exact test was used for comparison of the differences in age composition ratios of the 10 pathogens.

* Percentage (%) is the proportion of each bacteria pathogen among the 10 major pathogens.

# Percentage (%) is the proportion of each kind of pathogen under different age groups.

**Figure 2:**
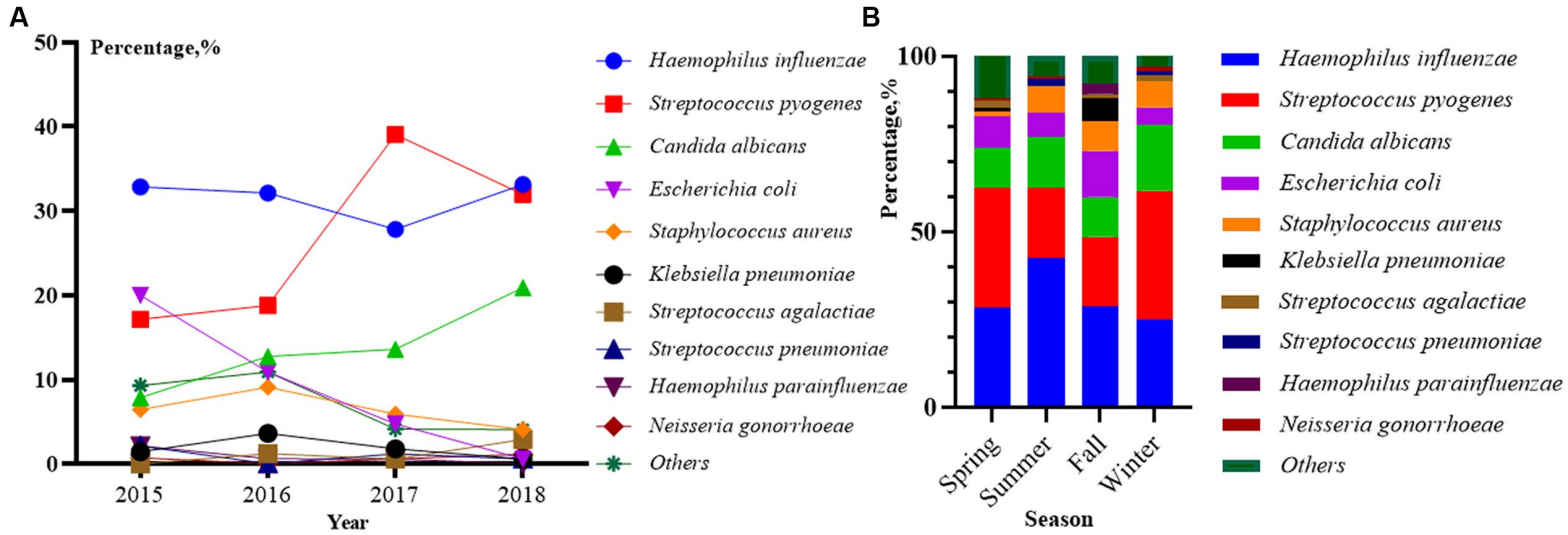
Distribution of top 10 pathogens among 646 outpatients by year **(A)** and season **(B)** from 2015 to 2018 (*p* < 0.01). *p* < 0.01 in **(A)** represents a significant difference in the overall composition ratio of the above 10 pathogens over 4 years. R × C fisher’s exact test was used for comparing whether there was a difference in the composition ratio of 10 pathogens in 4 years (Others were not included). p < 0.01 in **(B)** represents a significant difference in the overall composition ratio of the above 10 pathogens over four seasons. R × C fisher’s exact test was used for comparing whether there was a difference in the composition ratio of 10 pathogens in four seasons (Others were not included).

The year and season distribution of patients also differed significantly within the 10 pathogen classifications (*p* < 0.05, Figures 2A,B). From 2015 to 2018, *Haemophilus influenza*, *Streptococcus pyogenes* and *Candida albicans* were the main pathogens, and there was a gradual increase in the proportion. *Escherichia coli* showed a decreasing trend, and the other two pathogens accounted for a relatively stable proportion (Figure 2A). In terms of seasonal distribution (Figure 2B), *Haemophilus influenza* was the most common bacterial infection in summer at 42.53%. *Streptococcus pyogenes* were the most common bacterial infections in winter and spring, accounting for 36.59 and 34.04%, respectively. *Escherichia coli* and *Klebsiella pneumonia* were often seen in fall accounting for 13.17 and 6.59%, respectively. *Staphylococcus aureus* was rare in spring and evenly distributed in other seasons. *Candida albicans* were prevalent in all seasons, and data showed that the highest detection rate was in winter at 18.90%.

## Discussion

In the current study, we explored the patterns and epidemiological characteristics of genital inflammation among girls through an eight-year retrospective study of 49,175 patients. To best of our knowledge, this is the first large-sample and long-time-span retrospective observational study in China. Moreover, the consultation of genital inflammation has been increasing year by year in recent years, which indicated that genital inflammation among girls aged less than 18 might entail more attention from doctors, families, and society. The patterns of genital inflammation between genital inflammation in 0–18 girls and adults are significantly different. In adults, vulvovaginitis, cervicitis, and pelvic inflammation are common problems. Vulvovaginitis among adults is usually caused by bacterial, fungal, mycoplasma, chlamydia, and human papillomavirus (16). The most widespread issue among children and adolescents is non-specific vulvovaginitis (30,247, 61.51%). Lichen sclerosis, labial adhesion, bacterial vulvovaginitis, vulvovaginal candidiasis, vaginal foreign body, vulvar abscess, and STIs are in order, depending on the prevalence. Our result is consistent with western previous reviews about genital infection in children and adolescents and completely different from the spectrum of adult diseases (8, 17). Secondly, adult genital inflammation was closely associated with a history of sexual intercourse, abortion, and unclean cervical manipulation, with a higher proportion of endogenous infections than exogenous infections. STIs are relatively common diseases in adults (18). For children and adolescents, the presentence of first-visit (0.1%) for STIs was extremely low (51, 0.1%) in the current study. The history of our patients with STIs showed they did not have sexual life or sexual abuse. Some of their parents or grandparents have a similar infection. Some of them have been to the public bathroom or swimming pool. The possible reason for preschool girls with STIs is not due to sexual transmission in our study, which is quite different from the epidemic characteristics of girls in other countries. In the United States, approximately 20 million new STIs occur, half of the cases among adolescents aged 15–24 years (19). In all, our study described the whole picture of epidemiological characteristics and categories of girls’ genital inflammation in China, not only including vulvovaginitis caused by bacteria or fungi, but also vaginal foreign body and other factors.

The age distribution characteristic of genital inflammation was mainly concentrated in preschool girls in the current study. Hypoestrogenism, the anatomical proximity of the rectum, and delicate vulvar skin and vaginal mucosa are the physical features of preschool girls (13). Other different risk factors for children should also be considered, such as the long-time diaper use, improper urination habits, chemical irritants, poor hygiene, higher consumption of energy-dense, and high-glycemic-index foods (2, 20). Allergic problem is also a possible risk factor for the age distribution of genital inflammation. A multicenter study indicated that atopic dermatitis was observed most in the1-4 years’ groups (21). Previous studies indicated a strong association between allergic disease and lichen sclerosis (22, 23). Considering allergic factors are risk factors of lichen sclerosis, allergic problems should receive more attention in preschool girls. What’s more, psychological factor should also consider. Preschool girls in China were usually reared by mother or grandparents during 0–3 years old and went to the kindergarten during 3–6 years old. The previous study found that 3–6 years old children had high scores of separation anxiety disorder, which may attribute to their transition from family to kindergarten (24). We found a kind of special genital inflammation, a vaginal foreign body, was also common during this period. A prior study showed that patients with vaginal foreign bodies were between 1.5 and 14.8 years, with 3–10 years being the period of peak incidence (25), which is similar with our study. Hence, preschool girls require more careful care, more comfortable parenting pattern and necessary psychological referral and intervention.

The vaginal micro-flora is a complicated environment, composed of varying microbiological species in variable quantities and relative proportions. When this ecosystem gets disrupted, the vaginal epithelium is less protected, and vaginal infection sets in. Typically, vaginal infections are characterized by a shift in microbial communities that include a progressive replacement of certain *Lactobacillus species* by pathogenic or opportunistic microorganisms. This microbial shift can lead to different vaginal infections (26) The current study also explored common potential pathogens and the age distribution. The top five pathogens were *Haemophilus influenzae*, *Streptococcus pyogenes, Candida albicans, Escherichia coli and Staphylococcus aureus*. Earlier studies from various countries or regions showed *Streptococcus pyogenes* and *Haemophilus influenzae* were the two most identified pathogenic bacteria found in prepubertal girls with vulvovaginitis (11, 12, 27), which was consistent with our result. Previous study indicated AV can be frequently caused by *Group B streptococci*, *Escherichia coli*, *Staphylococcus aureus* (28). BV usually associated with several anaerobic or facultative bacteria, the most prevalent being: *G. vaginalis*; *Bacteroides* sp.etc. Another vaginal infection is VVC due to *Candida albicans*, *Candida glabrata*, and *Candida tropicalis*. The most prevalent form of vaginal infection in our study was belong to AV, because *Streptococcus pyogenes*, *Escherichia coli and Staphylococcus aureus* are three of the five common pathogens. AV is still known about its global epidemiology and implications, when comparing BV and VC, especially to young girls. Our result was in accordance with a previous study from Greece, which showed AV was more frequent in pubertal under-aged females, BV in reproductive age adult females (29). To adults, *Garderella vaginalis* appears to be the most virulent BV-associated anaerobe, which influences the biofilm formation (30). However, in the current study, *Garderella.* vaginalis has not been detected, which need further investigation. The VVC was mostly in 7–18 years old, especially in late adolescent girls (77.78%). The main pathogen was *C. albicans*. High estrogen levels and glycogen secretion in late adolescent girls changed the internal vaginal environment, such as pH values, and enhanced the adherence of fungi to cause VVC infection (28). In addition, the reason may also have been due to a stressful life in preparing for the College Entrance Examination, which may easily have caused VVC infection by impaired immunity. Moreover, VVC infection can be due to the growth and reproduction of fungi in a warm and humid environment, which also explains why VVC typically intensifies in July and August. Recurrence of VVC is also a problem for adolescent girls. In our study, follow-up times of girls aged 7–18 years old are more frequent than girls aged 0–6 years old. The high prevalence, substantial morbidity, and economic losses of recurrent vulvovaginal candidiasis exist not only in adults but also adolescents, which requires better solutions and improved quality of care for affected women (29).

The present study is the first retrospective study with a large sample size and comprehensive analysis exploring the spectrum of genital inflammation in pediatric and adolescent patients in China. The large sample size, long-time span, and wide range of disease conditions provide a better understanding of genital inflammation problems among Chinese girls. In addition, this study also analyzes common pathogenic data to supplement further data on the pathogens of pediatric female reproductive system diseases in China. However, the present study also has certain limitations. The basic demographic information obtained through electronic medical record data in this study was limited to pathogens detection and diagnosis, so the patients’ daily health awareness, behaviors and clinical presentation could not be further explored, and only preliminary etiological hypotheses could be proposed. In addition, certain pathogens (e.g., *Garderella*, viruses) cannot be grown in existing culture media, which hindered us from fully understanding the pathogens of genital inflammation among girls. In future, we will improve the methods to reveal the accurate relationship between genital inflammation and vaginal dysbiosis. Moreover, it is a single-center study in which the population was mostly from within Zhejiang Province, and multicenter studies in China would be investigated in future. This would be more representative of the overall situation of genital condition among girls in China.

## Conclusion

The patterns and epidemiological characteristics of genital inflammation at the first clinical visit in China have seldom been reported. Our study helps to fill this gap by examining the pattern of diagnoses among patients at the first clinical visit over an 8-year period in China. Overall, non-specific vulvovaginitis, lichen sclerosis, and labial adhesion were common issues for pediatric and adolescent patients with genital inflammation. The peak age of the first visit was 0–6 years old. The specific disease pattern varied among different age groups. Our study also reveals an increased number of genital inflammation patients in China over the decades, emphasizing the need for more public awareness, health education, healthcare services, and epidemiological research in this field.

## Data availability statement

The original contributions presented in the study are included in the article/Supplementary material, further inquiries can be directed to the corresponding authors.

## Ethics statement

The studies involving human participants were reviewed and approved by The study was approved by the Human Subjects Committees of the Children’s Hospital, Zhejiang University School of Medicine (approval number 2019-IBR-103). Written informed consent from the participants’ legal guardian/next of kin was not required to participate in this study in accordance with the national legislation and the institutional requirements.

## Author contributions

HG, MZ, and LS collected clinical data. HG and YZ did the conceptualization, design work, and wrote the original draft. YP, MZ, YQ, and SH contributed to the data analysis. SC, PS, KT, and CY supervised the research. All authors contributed to the article and approved the submitted version.

## Funding

This work was supported by the National Nature Science Foundation of Zhejiang (LY20H040011).

## Conflict of interest

The authors declare that the research was conducted in the absence of any commercial or financial relationships that could be construed as a potential conflict of interest.

## Publisher’s note

All claims expressed in this article are solely those of the authors and do not necessarily represent those of their affiliated organizations, or those of the publisher, the editors and the reviewers. Any product that may be evaluated in this article, or claim that may be made by its manufacturer, is not guaranteed or endorsed by the publisher.
