# Supplementary figures and images for: Patterns of pediatric and adolescent female genital inflammation in China: an eight-year retrospective study of 49,175 patients in China
Source: Front Public Health. 2023 Sep 4;11:1073886. doi: 10.3389/fpubh.2023.1073886 (PMC10506404; doi:10.3389/fpubh.2023.1073886)

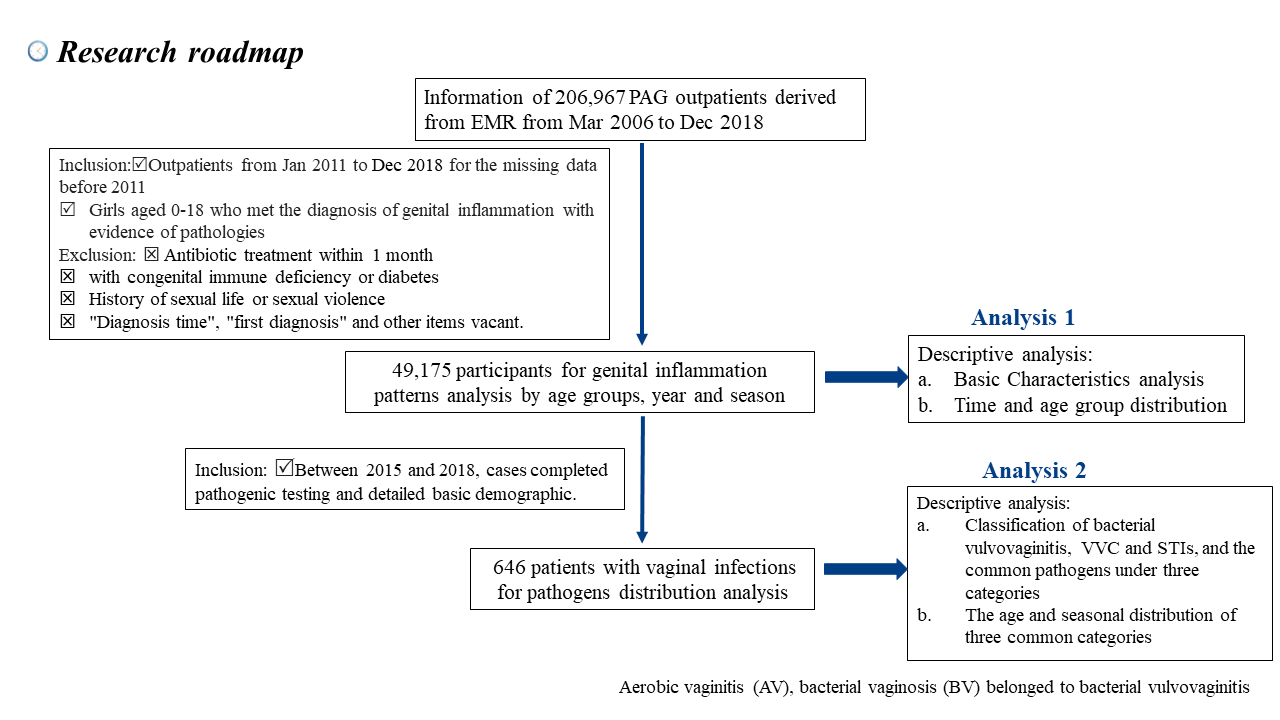

Supplement: Supplementary file 1 [file Image_1.tif]
